# Unveiling the Potential Neuroprotective Effect of Bioactive Compounds from Plants with Sedative and Mood-Modulating Properties: Innovative Approaches for the Prevention of Alzheimer's and Parkinson's Diseases

**DOI:** 10.2174/011570159X345397241210103538

**Published:** 2025-02-11

**Authors:** Silvia Piccirillo, Alessandra Preziuso, Tiziano Serfilippi, Giorgia Cerqueni, Valentina Terenzi, Vincenzo Lariccia, Simona Magi

**Affiliations:** 1 Department of Biomedical Sciences and Public Health, School of Medicine, University “Politecnica delle Marche”, Via Tronto 10/A, Ancona, 60126, Italy

**Keywords:** Phytobioactive compounds, neurodegeneration, oxidative stress, neuroinflammation, neuroprotection, Parkinson’s disease, Alzheimer’s disease

## Abstract

Neurodegenerative diseases like Alzheimer's disease and Parkinson's disease are severe disorders characterized by progressive neuron degeneration, leading to cognitive decline, motor dysfunction, and other neurological issues, significantly impairing daily life and the quality of life. Despite advancements in understanding these mechanisms, many aspects remain unclear, and current treatments primarily manage symptoms without halting disease progression. Multiple biological pathways are implicated in neurodegeneration, including oxidative stress, neuroinflammation, mitochondrial dysfunction, and aberrant protein folding. Given the multifactorial nature of neurodegenerative diseases, a neuroprotective approach targeting various mechanisms holds significant promise for prevention. Natural products derived from plants, animals, and fungi, known for their antioxidant and anti-inflammatory properties, show substantial potential in the prevention of neurodegeneration. Unlike synthetic compounds, bioactive compounds from these natural sources offer diverse targets due to their varied structures and biological activities. This review focuses on the potential of bioactive compounds from plants with sedative and mood-modulating effects in preventing and/or slowing down neurodegeneration.

## 1 INTRODUCTION

Neurodegenerative diseases, such as Alzheimer’s Disease (AD) and Parkinson’s Disease (PD), are debilitating disorders of the nervous system characterized by progressive degeneration of neurons, resulting in gradual loss of cognitive function, motor control, and other neurological processes [1]. This condition significantly impairs daily functioning with devastating effects on the quality of life [2, 3]. After extensive years of research, substantial evidence points to multifactorial conditions as the roots of these disorders. These include 1) abnormal protein folding leading to defective protein degradation and aggregation, 2) oxidative stress triggering free radical formation, 3) impaired bioenergetics and mitochondrial dysfunction, and 4) exposure to metal toxicity and pesticides [4-6]. Despite extensive research aimed at unravelling the molecular mechanisms behind neurodegeneration, many aspects of these pathologies remain enigmatic. The current interventions for neurodegenerative diseases primarily focus on symptom management, with real disease-modifying treatments still under exploration.

AD is a progressive neurodegenerative disorder that primarily affects brain function, leading to memory loss and cognitive decline [7]. It is characterized by the accumulation of abnormal protein deposits in the brain, including beta-amyloid plaques (Aβ) and neurofibrillary tangles composed of hyperphosphorylated Tau protein (pTau) [8]. These pathological alterations result in neuron degeneration and disruption of synaptic transmission, particularly in the regions of the brain responsible for memory and cognitive functions [9]. As the disease progresses, individuals may experience difficulties with language, problem-solving, and performing familiar tasks.

PD is the most common age-related motoric neurodegenerative disease characterized by the gradual deterioration of dopaminergic neurons located in the substantia nigra pars compacta (SNpc) and the abnormal aggregation and accumulation of α-synuclein (α-syn) in the form of Lewy bodies, which accumulate within neurons disrupting normal cellular function and contributing to the progression of PD [10]. This neuronal loss in the SNpc results in the depletion of dopamine, which is a neurotransmitter critical for regulating movement [3, 11]. Consequently, the clinical features traditionally associated with PD are motor symptoms, including tremors, rigidity, bradykinesia, and postural instability. However, PD is now recognized to be a complex disorder involving a wide range of non-motor manifestations that contribute to disability.

Given the multifactorial nature of pathological mechanisms associated with neurodegeneration, adopting a neuroprotection approach that targets various mechanisms could be a promising strategy for the prevention of neurodegenerative diseases. Over the past few years, there has been a surge of interest in natural products derived from plants, animals, and fungi, along with their bioactive compounds, due to their inherent antioxidant and anti-inflammatory properties, which hold significant potential in preventing and/or delaying neurodegeneration. Interestingly, bioactive compounds offer a wider range of targets compared to chemical compounds due to their varied structures and biological activities [12].

**Fig. (1):**
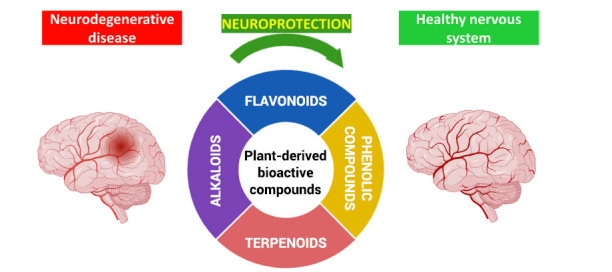
Progression of neurodegenerative disorders.

In this review, we focused on the potential benefits of bioactive compounds extracted from plants possessing sedative and mood-modulating effects in the context of neurodegenerative disorders. These compounds refer to chemical substances found in certain plant species that have physiological effects on the body, particularly in promoting relaxation, reducing anxiety, and improving mood. Although these plants are renowned for their calming and relaxing effects, there is growing attention to their potential neuroprotective properties against neurodegeneration. These compounds, encompassing flavonoids, terpenoids, alkaloids, and phenolic compounds, are significant for their capacity to reduce oxidative stress and inflammation, both of which are implicated in the onset and progression of neurodegenerative disorders, such as AD and PD (Fig. **1**) [13].

## 2 MECHANISMS AND POTENTIAL TARGETS OF NEURODEGENERATION

Neurodegenerative disorders encompass a complex interplay of various pathological mechanisms, each contributing to the progressive decline in neuronal function and viability. Among these mechanisms, oxidative stress arises from an intricate imbalance between the production of free radicals and the capacity of antioxidant defenses within cells [14]. Free radicals, such as reactive oxygen species (ROS) and reactive nitrogen species (RNS), are highly reactive molecules generated as natural byproducts of cellular metabolism, especially within mitochondria, as well as in response to environmental stressors [15]. Under normal circumstances, cells possess an efficient repertoire of antioxidant enzymes, including superoxide dismutase, catalase, and glutathione peroxidase, along with non-enzymatic antioxidants like vitamins C and E, which work synergistically to neutralize these harmful radicals and maintain redox homeostasis [16]. However, in the context of neurodegenerative diseases, this delicate balance is disrupted, resulting in an overwhelming accumulation of free radicals that surpass the scavenging capacity of endogenous antioxidants [14, 17]. Consequently, these unchecked radicals indiscriminately target cellular macromolecules, including lipids, proteins, and nucleic acids, leading to oxidative damage and functional impairment [18]. Moreover, oxidative stress serves as a powerful catalyst, amplifying neurodegenerative processes by triggering a cascade of events that worsens cellular dysfunction and promotes cell death [14]. For instance, oxidative modification of proteins can promote misfolding and aggregation, contributing to the formation of toxic protein aggregates that characterize both AD and PD pathogenesis [17, 19-22]. Additionally, oxidative stress can further exacerbate mitochondrial dysfunction, leading to impaired energy metabolism and increased ROS production, thus perpetuating a vicious cycle of cellular damage and dysfunction [23-25]. Dysfunction in mitochondrial activity, characterized by compromised energy production and increased ROS generation, significantly contributes to neurodegenerative processes, including metabolism dysfunction [17, 26]. However, the consequences of mitochondrial dysfunction extend beyond impaired energy metabolism and oxidative stress. Indeed, since mitochondria are crucial regulators of calcium (Ca^2+^) homeostasis, their dysfunction can disrupt intracellular Ca^2+^ signaling, leading to neuronal excitotoxicity and cell death [17, 27]. Moreover, dysfunctional mitochondria are unable to adequately regulate apoptotic pathways, resulting in increased susceptibility to programmed cell death [28]. Another critical factor involved in the pathogenesis of neurodegenerative disorders is neuroinflammation. It is characterized by the activation of various immune cells within the Central Nervous System (CNS), including microglia and astrocytes, in response to pathological stimuli [29]. This inflammatory response is a double-edged sword, as while it is initially intended to protect the brain from harmful insults, prolonged or dysregulated activation can lead to detrimental consequences [30]. Indeed, prolonged activation of microglia and astrocytes triggers the release of pro-inflammatory cytokines, chemokines, and ROS, which not only exacerbate neuronal damage but also disrupt the delicate balance of neurotransmitters and neurotrophic factors essential for physiological neural function [31]. This disruption in neural signaling pathways further fuels the progression of neurodegeneration, creating a vicious cycle of neuronal injury and inflammation [32, 33]. Furthermore, neuroinflammation is intricately linked with oxidative stress and protein aggregation, contributing to amplifying the detrimental effects of neuroinflammation on neuronal health and accelerating disease progression [34].

Considering the multifaceted nature of neurodegeneration, pursuing a strategy that targets multiple mechanisms of action holds promise for both preventing and treating neurodegenerative diseases. It is noteworthy that bioactive compounds act directly on these pathological mechanisms, offering neuroprotective effects by attenuating oxidative stress, restoring mitochondrial function, and dampening inflammatory responses.

## 3 PLANT-DERIVED BIOACTIVE COMPOUNDS

Phytobioactive compounds are plant-derived substances that offer protection against various diseases, yet they are not essential components of our diet. These substances, characterized by unique structural and functional properties, are commonly known as phytochemicals or secondary metabolites of plants [35]. These compounds include flavonoids, terpenoids, alkaloids, and phenolic compounds.

Polyphenols are a diverse group of phytochemicals found in plant-based foods, such as fruits, vegetables, nuts, seeds, and beverages like tea and red wine. They are characterized by their multiple phenol rings and can be categorized into subclasses, such as flavonoids, phenolic acids, and stilbenes [36]. One of the key properties of polyphenols is antioxidant activity, which involves scavenging free radicals and enhancing the expression of antioxidant enzymes, such as γ-glutamylcysteine synthetase (γGCS), glutathione peroxidase (GPX), superoxide dismutase (SOD), and glutathione reductase (GSH). This activity helps to reduce the risk of various chronic diseases, such as neurodegenerative disorders [37]. Moreover, polyphenols exhibit a range of other beneficial effects beyond their antioxidant properties, including anti-inflammatory activity [38]. Different polyphenolic compounds may vary in their antioxidant and anti-inflammatory capacity, depending on factors, such as their chemical structure, concentration, and bioavailability [12].

Flavonoids, a subgroup of polyphenolic compounds abundantly found in fruits (apples, berries, cherries [39]), vegetables (onions [39]), and medical plants (chamomile [40]), have garnered considerable attention for their potential neuroprotective properties [41]. Flavonoids exhibit anti-inflammatory properties by inhibiting the production and release of pro-inflammatory mediators, such as cytokines and chemokines, and suppressing the activation of microglia and astrocytes, thereby slowing neurodegeneration. Flavonoids also modulate key molecular targets involved in neurodegenerative processes, including the inhibition of Aβ aggregation in AD and the modulation of α-syn accumulation in PD [42, 43]. These compounds also promote neurogenesis and enhance synaptic plasticity, which is essential for learning and memory [44, 45]. Their antioxidant and anti-inflammatory properties help protect neurons from oxidative damage and neuroinflammation while also supporting neuronal survival by influencing signaling pathways related to apoptosis, neurogenesis, and synaptic function [46]. Beyond their antioxidant and anti-inflammatory actions, phenolic compounds are also involved in the inhibition of toxic protein aggregation in both AD and PD [47] and in the promotion of neuronal survival by modulating signaling pathways involved in apoptosis, neurogenesis, and synaptic plasticity [48]. Research highlights the neuroprotective actions of flavonoids, such as nobiletin, fisetin, and cocoa, in neurodegenerative disorders [40]. Nobiletin, derived from citrus peels, shows promise in treating AD and PD by reducing Aβ plaque and pTau accumulation, alleviating oxidative stress, and improving both motor and cognitive functions [49]. Fisetin, found in strawberries, exerts a neuroprotective role in PD by boosting antioxidant defenses, supporting cognitive function, and increasing dopamine levels [50]. Cocoa, rich in flavonoids like flavan-3-ols, helps mitigate Aβ-induced neurotoxicity, preserves cell viability, and supports neuronal network integrity. Its antioxidant and anti-inflammatory properties make it a potential candidate for attenuating neurodegeneration [51]. Additionally, cocoa offers cardiovascular benefits by reducing arterial stiffness, lowering triglycerides, enhancing nitric oxide synthase activity [52], and reducing oxidative stress and platelet reactivity [53].

Terpenoids, also known as terpenes, are aromatic compounds found in plants (salvia) [54], which possess diverse pharmacological properties. They have demonstrated promise in enhancing cognitive function, reducing neuroinflammation, and protecting against neuronal damage in neurodegenerative diseases. In AD, terpenoids may help preserve neuronal function and prevent the formation of Aβ plaques and tau tangles [55]. Similarly, in PD, they could protect dopaminergic neurons in the substantia nigra, potentially slowing the progression of the disease [56]. Another property of terpenoids is the modulation of neurotransmitter levels and activity. For instance, they may enhance cholinergic function in AD by inhibiting acetylcholine breakdown [57] and improve motor symptoms in PD by affecting dopamine levels or receptor sensitivity [58]. Notably, terpenoids can cross the blood-brain barrier, allowing them to exert their neuroprotective effects directly within the central nervous system, making them valuable for treating conditions like AD and PD [59].

An important terpenoid-based treatment is derived from *Astragalus membranaceus* (*A. membranaceus*), a plant well-known for its neuroprotective properties. Studies indicate that *A. membranaceus* significantly enhances recovery from hemorrhagic stroke by reducing brain edema and improving patient outcomes through its antioxidative and anti-inflammatory effects [60]. Additionally, Astragaloside IV (AS-IV), a compound from *A. membranaceus*, has shown neuroprotective benefits across several neurological disorder models. AS-IV reduces blood-brain barrier permeability, lymphocyte infiltration, neuroinflammation, apoptosis, and oxidative stress. In AD models, it inhibits Aβ production and plaque formation, while in PD, it promotes neuron survival by enhancing antioxidant defenses [61].

Alkaloids are nitrogen-containing compounds found in many plants, and most of them have definite pharmacological properties [35]. Their antioxidative properties make them promising candidates for treating neurodegenerative diseases by addressing oxidative stress. Studies have shown that alkaloids demonstrate anti-inflammatory properties by inhibiting various pro-inflammatory protein complexes [62], including NF-κB, ERK1/2, Akt, and STAT1, as well as inflammatory mediators like prostaglandin E2, nitric oxide, cytokines, and chemokines [63, 64]. Interestingly, alkaloids can improve the pathophysiology of neurodegenerative disorders by functioning as inhibitors of monoamine oxidase (MAO), acetylcholinesterase, and butyrylcholinesterase and by acting as N-methyl-D-aspartate (NMDA) antagonists, as well as agonists of muscarinic and adenosine receptors [65].

The diverse mechanisms of action of these bioactive compounds make them appealing candidates for preventing neurodegenerative diseases.

**Fig. (2):**
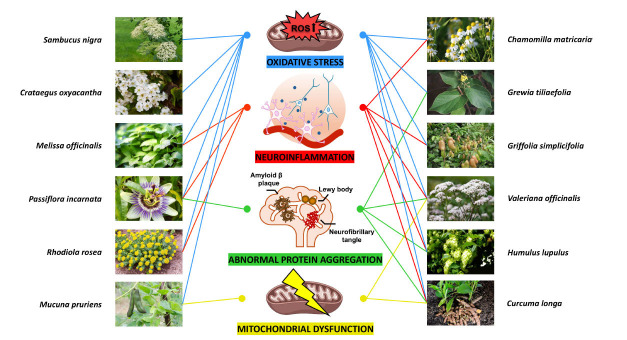
The diverse mechanisms of action of these bioactive compounds.

In the following paragraph, we will discuss how natural products and their bioactive compounds show promise in alleviating neurodegenerative processes (Fig. **2**).

## 4 NEUROPROTECTIVE EFFECTS OF NATURAL PRODUCTS AND THEIR BIOACTIVE COMPOUNDS IN AD AND PD

### 4.1 *Griffonia simplicifolia*

*G. simplicifolia* is a climbing leguminous plant belonging to the family of Fabaceae, native to West Africa, especially in Ghana, Liberia, Togo, and the Ivory Coast. Phytochemical analyses demonstrated that this plant contains several active compounds as well as non-proteinogenic amino acids, such as 5-hydroxytryptophan, a known precursor of serotonin [66]. *G. simplicifolia* may be considered an effective natural alternative to selective serotonin reuptake inhibitors and synthetic antidepressants for the treatment of mood disorders, anxiety, and depression [67], and it is also used as a secondary treatment for sleep disorders and appetite [68, 69]. Furthermore, *G. simplicifolia* exerts several biological actions, including antioxidant, anti-inflammatory, antibacterial, and anticancer activities, due to tannins, flavonols, and carbolide alkaloids [70]. Gueyraud *et al*. explored the antioxidant potential of the aqueous leaf extract of *G. simplicifolia* [71]. They first demonstrated that the viability of neural cells was not compromised with an extract concentration of up to 800 μg/mL, while the concentration of 20 μg/mL was able to protect neurons and astrocytes from oxidative stress induced by hydrogen peroxide [71]. Regarding the seed extracts, a metabolomic study on different used solvents highlighted acetone and ethanol as optimal for the extraction of flavonoids and flavan-3-ols, while microwave-assisted extraction displayed enhanced effectiveness in extracting N-containing compounds, including 5-hydroxytryptophan. Furthermore, this analysis demonstrated that the hydroalcoholic extracts exhibited the highest radical scavenging and metal-reducing antioxidant power [72].

### 4.2 *Sambucus nigra*

*S. nigra* is an herbaceous species of the Caprifoliaceae family, and it spontaneously grows in Europe, West Asia, and North America. Although its leaves and stems are toxic due to their content of cyanogenic glycosides (sambunigrin and prunasin) and m-hydroxysubstituted glycosides (zierin and holocalin), which, after hydrolysis, can release cyanide [73], the elderflower and fruits are used as herbal medicine and for food purposes [74-76]. Quercetin, rutin, and kaempferol, potent flavonoids, and chlorogenic, caffeic, and protocatechuic acids, known as hydroxycinnamic acids, give elderflower its antibacterial [77], immunomodulatory [78], diuretic and anti-diabetic properties [79]. The antioxidant and protective potential of elderflower were confirmed in the human neuroblastoma SH-SY5Y cell line [80]. The *S. nigra* flower extract also exhibited the ability to regulate mTORC1 signaling activity, and therefore, it positively regulated the pathogenic effects of molecular mechanisms involved in neurodegenerative diseases [80].

### 4.3 *Grewia tiliaefolia*

*G. tiliaefolia* is a subtropical tree specifically found in the Himalayan tract from Jammu to Assan, the central Vindya range, and the Western and Eastern Ghats of India [81]. In Indian traditional medicine, the plant is used as an astringent, expectorant, antipruritic, and aphrodisiac [82]. The *Grewia* genus consists of 150 species and is a member of the family *Tiliaceae* found in tropical and subtropical areas; about 40 of these species are found in India [83]. The methanol extract of *G. tiliaefolia* has shown significant free radical scavenging properties and an electron-donating ability and, therefore, can be considered an effective antioxidant [82]. Since different antioxidants have demonstrated protective effects against the neurodegenerative spiral induced by Alzheimer's redox imbalance [25, 84], methanol extracts of *G. tiliaefolia* may be protective as well. Leaf extracts also showed the ability to inhibit cholinesterase enzymes. Notably, polar extracts (methanol and water) showed higher inhibition compared to non-polar solvent extracts. Various compounds of methanol extract, such as vitexin, vitexin-4-O-glucoside, 3-O-methyl ellagic acid, isovitexin, and nitidanin, demonstrated the ability to interact with various docking sites present on the AchE enzyme. Of note, vitexin showed significant and dose-dependent inhibition of both AchE and BchE [82, 83]. Moreover, a methanol extract of *G. tiliaefolia* managed to protect Neuro2a cells from the cytotoxic effects of Aβ_25-35_ while also restoring cell viability [82]. Malar *et al*. reported that Aβ_25-35_ leads to an increase in fluorescence intensity, indicating the formation of oligomers and aggregates. Treatment with methanol extract of *G. tiliaefolia* managed to reduce the intensity of fluorescence similarly to galantamine (50 µM). Therefore, the methanol extract managed to reduce Aβ_25-35_ aggregation and fibril formation [82].

### 4.4 *Passiflora incarnata*

*P. incarnata*, commonly known as the passionflower, belongs to the Passifloraceae family, and it is a flowering plant renowned for its medicinal properties. *P. incarnata* is native to the southeastern United States, particularly in regions like Florida and Texas, as well as parts of Central and South America. The main constituents of the leaves of *P. incarnata* are flavonoids, maltol, and indole alkaloids [85]. Historically, the pharmacological investigations of *P. incarnata* primarily focused on its sedative, anxiolytic, and anticonvulsant impacts on the CNS, alongside its potential to aid in the treatment of addiction [86-89]. Later on, further research highlighted the neuroprotective properties of *P. incarnata*, shedding light on its potential to mitigate the loss of neurons in PD and AD, thereby preserving cognitive function and motor abilities in affected individuals [90]. It has been reported that the most promising neuroactive substances are flavonoids, such as chrysin, vitexin, isovitexin, orientin, isoorientin, apigenin, and kaempferol, as well as indole alkaloids [91]. In a study conducted by Kim *et al*., vitexin was identified as the active compound in the extract of *P. incarnata*, showing a diverse array of biological functions, including antioxidant, anti-inflammatory, and anti-AD properties [92]. Notably, the study reported substantial enhancements in neurocognitive function and reductions in pTau/Tau levels following treatment with *P. incarnata* extract [92]. *P. incarnata* also demonstrated antiparkinsonian and memory-enhancing effects, probably due to its high phenolic and flavonoid content [90]. Specifically, the butanol extract of *P. incarnata* exhibited significant reductions in haloperidol-induced catalepsy, tacrine-induced jaw movements, and the overproduction of free radicals [90]. A recent study reported that the extract of *P. incarnata* significantly impacts dopaminergic neurotransmission and dopamine metabolism in the CNS [93]. In this regard, an increased level of dopamine and its metabolites was observed in the spinal cord following treatment with an extract of *P. incarnata*, suggesting its potentially beneficial effect on PD, which is characterized by a deficit in dopaminergic neurotransmission [93].

### 4.5 *Crataegus oxyacantha*

*C. oxyacantha*, also known as Hawthorn, is a shrub of the Rosaceae family that can be found in the hilly areas of Himachal Pradesh and Kashmir [94]. This plant is a rich source of triterpenic acids, ursolic acid, oleanolic acid, polyphenols, flavonoids, glycosides, and other important organic molecules [95]. Due to this composition, *C. oxyacantha* is used to treat many cardiovascular diseases [96, 97], as well as hypertension, arthritis, and hyperlipidaemia [98]. Another important role of *C. oxyacantha* is in the prevention of neurodegenerative disorders, such as AD and dementia, as well as attention deficit disorder and anxiety [99]. Indeed, extracts of this fruit can be used as natural antimicrobial and antioxidant preparations [100]. Different studies have demonstrated the ameliorative role of *C. oxyacantha* in cognitive impairment and brain oxidative damage [101] and its possible use in AChE and BChE inhibitions [99]. *C. oxyacantha* also demonstrates an ability to enhance learning and memory. This is observed through a reduction in the transfer latency time in rats with scopolamine-induced amnesia on an elevated maze. Furthermore, it helps restore the balance of oxidative stress markers in the brain that are disturbed due to the administration of scopolamine. Additionally, it inhibits serotonergic transmission while promoting dopaminergic transmission [102]. In conclusion, *C. oxyacantha* is a rich source of neurologically potent organic molecules, which play a vital role in the attenuation of neurodegeneration and its related nervous system and neuroprotective effect.

### 4.6 *Valeriana officinalis*

*V. officinalis*, a perennial herb belonging to the Caprifoliaceae family, is widely distributed across America, Europe, and Asia [103]. Among approximately 200 identified species, *V. officinalis* is particularly notable for its medicinal applications [104]. The bioactive constituents of *V. officinalis*, including valerenic acid, valerenal, and various antioxidants, contribute to its efficacy as a natural remedy for anxiety and sleep disorders [105]. These compounds interact with neurotransmitter receptors in the brain, such as gamma-aminobutyric acid (GABA) receptors, promoting relaxation and reducing nervous tension. In addition to its traditional use as a sedative, recent research has explored its potential in addressing neurodegenerative diseases like AD and PD [106]. Studies indicate that valerian extracts may possess neuroprotective effects by mitigating oxidative stress and inflammation, both of which are implicated in the progression of these pathological conditions. *V. officinalis* boasts a rich chemical composition, comprising over 150-200 chemical constituents [107]. Among these, flavonoids renowned for their effects on the CNS, as well as lignans with antioxidative and vasorelaxant properties [108]. De Oliveira and colleagues demonstrated that, in an *in vitro* model of PD, the aqueous extract of *V. officinalis* provides cytoprotection against rotenone-induced apoptosis in SH-SY5Y cells, suggesting a potential neuroprotective effect. This outcome is attributed to the antioxidant properties associated with lignans found in *V. officinalis*. Indeed, the generation of ROS and subsequent oxidative stress resulting from the inhibition of mitochondrial complex I induced by rotenone seem to play a significant role in the PD model based on rotenone toxicity [109]. Another study conducted by Malva and colleagues found that *V. officinalis* has been associated with a GABAergic mechanism [110]. This study aimed to assess its neuroprotective potential against Aβ _25-35_ toxicity in primary rat hippocampal neurons. It was found that *V. officinalis* extract prevented neuronal injury induced by Aβ_25-35_, probably by inhibiting excess Ca^2+^ influx and membrane peroxidation. These findings highlighted the neuroprotective properties of *V. officinalis* extract against Aß toxicity, offering potential applications in aging and neurodegenerative disorders [110]. Marawne and colleagues investigated the effects of *V. officinalis* on glial cells under conditions mimicking neurodegenerative inflammation commonly observed in diseases, such as AD and PD [111]. In particular, they observed that the alcoholic extract of *V. officinalis* reduced microglial activation, NO production, and gene expression of Tumor Necrosis Factor α (TNF-α) and induced nitric oxide synthase (iNOS). Notably, lower concentrations of the extract mitigated inflammation, while higher doses promoted cell survival. Moreover, the secondary metabolites of *V. officinalis*, including valeric acid and valepotriates, were associated with relaxing, sleep-inducing, and neuroprotective properties. Specifically, valerenic acid exhibited potential in mitigating neurodegeneration in a mouse model of PD, potentially preserving dopaminergic neurons and restoring motor function. Additionally, valproic acid showed anti-inflammatory effects, reducing pain responses in mice and safeguarding against oxidative stress [111].

### 4.7 *Rhodiola rosea*

*R. rosea*, commonly known as the golden or arctic root, is a perennial flowering plant native to the Arctic regions of Europe, Asia, and North America. This plant belongs to the Crassulaceae plant family, specifically to the subfamily Sedoideae and the genus *Rhodiola* [112]. This botanical plant has been traditionally used to invigorate the nervous system, alleviate depression, improve work performance, and prevent altitude sickness. Among the various species within the *Rhodiola* genus, *R. rosea* has garnered considerable attention for its phytochemical composition and toxicological properties. The root of *R. rosea* contains approximately 28 compounds, with salidroside (rhodioloside), rosavins, and p-tyrosol, which are believed to exhibit the most significant beneficial effects. Research suggests that ingestion of *R. rosea* can enhance cognitive function, reduce mental fatigue, mitigate free radicals, boost endurance performance, and improve learning and memory [112]. Furthermore, there is a growing interest in the potential of *R. rosea* to mitigate neurodegenerative conditions like AD [113]. Its anti-inflammatory properties and neuroprotective abilities present promising avenues for exploring treatments for AD. Lee and colleagues investigated the anti-inflammatory and neuroprotective properties of constituents from *R. rosea* in both microglial and neuronal cells [112]. Microglia, known for their pivotal role in inflammatory reactions, are implicated in neurodegenerative pathologies like AD and PD, where their activation contributes to the production of harmful ROS and proinflammatory cytokines. Studies reported that *R. rosea* effectively inhibits the production of NO and the expression of iNOS in microglial cells stimulated with lipopolysaccharides (LPS), a known inflammatory stimulus. Additionally, *R. rosea* treatment reduced the levels of proinflammatory cytokines TNF-α, IL-1β, and IL-6 induced by LPS in microglial cells. Another study observed that oral administration of *R. rosea* extract decreased iNOS and proinflammatory cytokine responses in both the kidney and prefrontal cortex in mice, indicating its potential role in suppressing inflammation in the CNS. Furthermore, *R. rosea* demonstrated protective effects against L-glutamate-induced neurotoxicity in cortical neurons by inhibiting JNK and p38MAPK activation, underlining its neuroprotective potential against neurodegenerative diseases [112]. The pretreatment effects of *R. rosea* extract on cognitive dysfunction, oxidative stress, and hippocampal neuron injury in a rat model of AD were investigated by Qu *et al*. [114]. This study focused on the potential of *R. rosea* to exert a protective effect against AD-related deficits, particularly by reducing oxidative stress and improving cognitive function and neuronal damage. These findings suggest that *R. rosea* could potentially mitigate AD-associated cognitive deficits through its antioxidative properties.

### 4.8 *Humulus lupulus*

*H. lupulus*, commonly known as hops, is a perennial climbing plant known for its role in brewing beer, originating from Europe, Asia, and North America. With its distinctive cones, this member of the Cannabaceae family imparts bitterness, flavour, and aroma to the beverage [115]. Beyond brewing, hops have been used for centuries in traditional medicine for their sedative properties. Recently, *H. lupulus* has gained attention for its potential neuroprotective effects against neurodegenerative diseases [116]. Several studies have suggested that certain compounds found in hops, such as xanthohumol and its derivatives, possess antioxidant and anti-inflammatory properties that could help mitigate the progression of these debilitating conditions [116]. Additionally, hop extracts have shown promise in modulating neurotransmitter levels and improving cognitive function in experimental models of neurodegeneration [117]. A recent study conducted by Palmioli *et al*. explored the impact of hop extracts on the aggregation and toxicity of synthetic Aβ_1-42_ peptides [118]. Their investigation revealed the antioxidant capacity of hop extracts and their ability to promote autophagy, facilitating the clearance of amyloid aggregates. This study investigated the antioxidant characteristics of hop extracts, by testing them on SH-SY5Y cells to evaluate their antioxidant effects under oxidative stress conditions. Notably, hop extracts were found to not only inhibit the aggregation of Aβ_1-42_ but also to shield against Aβ-induced toxicity *in vitro*. Additionally, the polyphenols found in hop extracts emerged as potent anti-amyloidogenic agents, showing significant antioxidant properties [118]. Another study reported that the natural product extracted from *H. lupulus* inhibits monoacylglycerol lipase activity, reduces neurodegeneration, prevents neuroinflammation, and improves cognitive functions in AD animals [119].

### 4.9 *Chamomilla matricaria*

*C. matricaria* is a small flowering herbaceous plant belonging to the Asteraceae family [120]. Native to Europe, Asia, and North Africa, *C. matricaria* is characterized by its delicate, daisy-like flowers with white petals and yellow centers. This aromatic plant has a long history of use in various cultures for its medicinal, culinary, and cosmetic purposes [120]. *C. matricaria* is recognized for its calming and soothing properties, making it a popular ingredient in herbal teas, skincare products, and natural remedies. In addition to its traditional uses, *C. matricaria* has garnered attention for its potential beneficial effects in neurodegenerative diseases. Studies suggest that *C. matricaria* possesses antioxidant and anti-inflammatory properties, which may have neuroprotective effects [121]. These characteristics are due to the rich chemical composition of *C. matricaria*, such as α-bisabolol, chamazulene, bisabolol oxide A and B, matricin, and flavonoids like apigenin and luteolin [122]. In particular, α-bisabolol is renowned for its anti-inflammatory, antimicrobial, and skin-soothing properties, chamazulene possesses anti-inflammatory and antioxidant effects, and flavonoids contribute to the antioxidant properties of *C. matricaria,* and it may help alleviate inflammation and promote relaxation [123]. In a recent study conducted by Alahmady *et al*., the neuroprotective properties of *C. matricaria* were investigated in the context of AD [124]. It was found that, in rats exposed to aluminum chloride to induce cognitive impairment, an essential oil extracted from *C. matricaria* exhibited neuroprotective effects on the cholinergic system by increasing acetylcholine (ACh) levels and reducing inflammatory cytokines and apoptosis markers [124]. Moreover, *C. matricaria* extract exhibited antioxidant properties by protecting the rat brain from the increased oxidative stress caused by scopolamine administration, which is known to impair long-term potentiation [125]. These findings suggest that chamomile may offer a promising, cost-effective, and safe alternative to conventional AD therapy.

### 4.10 *Melissa officinalis*

*M. officinalis*, commonly known as lemon balm, is a perennial herbaceous plant belonging to the Lamiaceae family [126]. With a rich history dating back centuries, lemon balm has been revered for its medicinal properties and aromatic qualities. Native to the Mediterranean region and parts of Asia, this herb is now cultivated worldwide [126]. Rich in bioactive compounds like rosmarinic acid, flavonoids, and essential oils, lemon balm has been traditionally used to alleviate stress, aid digestion, and promote skin health [127-129]. Ongoing research explores its potential as an anti-inflammatory, antioxidant, and neuroprotective agent [129]. A study conducted by Ozarowski *et al*. examined the effects of prolonged administration of a standardized extract of *M. officinalis* on memory impairment induced by scopolamine in rats [130]. The study found that *M. officinalis* extract, containing rosmarinic acid, enhanced long-term memory in rats without affecting memory impairment [130]. Interestingly, the effects of the *M. officinalis* extract were studied in patients with mild to moderate AD [131]. It was observed that AD-affected patients showed significant cognitive benefits after 16 weeks of treatment with *M. officinalis* extract. Additionally, *M. officinalis* essential oil was found to positively affect agitation levels and the quality of life in patients with severe dementia [131]. *M. officinalis* has also been studied for the treatment of PD. In this regard, Martins and colleagues investigated the potential of *M. officinalis* extract in reducing manganese (Mn)-induced oxidative stress in mice, which can lead to neurodegenerative disorders resembling PD [132]. Of note, treatment with *M. officinalis* extracts significantly enhanced antioxidant enzyme activities and attenuated oxidative damage in the brain by replenishing the functions of superoxide dismutase and catalase enzymes, suggesting its potential antioxidative properties in mitigating Mn-induced oxidative stress [132].

### 4.11 *Curcuma longa*

Curcumin is the major component of *C. longa*, belonging to the Zingiberaceae family, which is a perennial herb widely cultivated in tropical regions of Asia [133]. Traditionally, curcumin has been used over the centuries for cooking as well as a phytotherapeutic agent [134]. Curcumin helps to maintain the homeostasis of the inflammatory system, improves the clearance of toxic aggregates from the brain, and induces antioxidant response elements [135-137]. However, the activity of curcumin is limited due to its low water solubility, fast biological metabolism, and limited blood-brain barrier (BBB) permeabilization [138]. To overcome this problem, various curcumin delivery systems have been developed based on nanoparticles. In PD drosophila and mice models, curcumin nanopreparation showed some anti-parkinsonism effects [139]. Specifically, *in vitro* results demonstrated that both α-syn oligomerization and fibrillation were significantly inhibited by curcumin, and that toxicity, oxidative stress, and cell death caused by rotenone were reduced [139]. Furthermore, studies reported that curcumin ameliorates behavioral damage in various AD models [140]. Curcumin is able to reverse neurotoxic damage induced by Aβ by preventing its aggregation and tau protein hyperphosphorylation, increasing mitochondrial ATP, and enhancing cytochrome oxidase activity [141].

### 4.12 *Mucuna pruriens*

*M. pruriens*, commonly known as velvet beans, belongs to the Fabaceae family and is native to India and the southern part of China [142]. It possesses valuable medicinal properties, including neuroprotective and antioxidant actions [143]. The major phenolic constituent of *M. pruriens* beans is the dopamine precursor, levodopa [144], which may help in ameliorating different types of free radical-mediated diseases like diabetes, arthritis, aging, and various other neurological disorders, such as PD [142]. Interestingly, the seeds of *M. pruriens* are rich in NADH and coenzyme Q10, which have been observed to exert beneficial effects in PD [145]. Animals treated with *M. pruriens* demonstrated significant improvement in behavioral tests along with reduced nerve damage and neuronal loss [146]. These findings suggest that *M. pruriens* could serve as an effective complementary treatment for PD, potentially enhancing the quality of life of the individuals affected by this disease [146]. Furthermore, treatment with the methanolic extract of *M. pruriens* significantly reversed memory deficits caused by ischemia [147]. This finding underscores its potential as a therapeutic option for severe brain injuries and neurodegenerative diseases.

## 5 METHODS

To select papers for this narrative review, we performed searches on PubMed and Google Scholar. The date of the most recent search was 21^st^ May, 2024. The following terms were used in the searches: Alzheimer, Alzheimer’s disease, Parkinson, Parkinson’s disease, oxidative stress, mitochondrial dysfunction, protein folding, sedative plants, mood-modulating plants, neuroprotection, neurodegeneration, phytobioactive compounds, *Griffonia simplicifolia, Sambucus nigra, Grewia tiliaefolia, Passiflora incarnata, Crataegus oxyacantha, Valeriana officinalis, Rhodiola rosea, Humulus lupulus, Chamomilla matricaria, Melissa officinalis, Curcuma longa,* and *Mucuna pruriens*.

## 6 LIMITATIONS AND CHALLENGES ASSOCIATED WITH THE USE OF NATURAL PRODUCTS AND THEIR BIOACTIVE COMPOUNDS IN AD AND PD

As detailed in the present work, there is a large number of preclinical studies investigating and corroborating the beneficial effects of natural products and their bioactive compounds on AD and PD. However, we also have to take into account the various limitations of a phytotherapeutic approach.

Indeed, plant extracts usually contain different active biochemical compounds, each capable of exerting activity on various molecular targets. Furthermore, nutraceuticals derived from plants usually have a low and highly variable bioavailability, while herbal drug composition is usually inconsistent [148]. Inter-individual variability is another challenge to the effective use of plant extracts in clinical practice; age, sex, microbiome, genetic differences, and diet can influence the organism's response to such nutraceuticals [149]. Therefore, each promising compound should be isolated and purified to a high degree or artificially synthetized. Its individual efficacy must then be evaluated first on cellular models, then on animal models, and, ultimately, in human trials. Lastly, plant extracts and natural compounds can negatively interact with a vast array of drugs [150], potentially reducing their absorption (and therefore their bioavailability), their elimination or activation (by inhibiting their secretion or their metabolism), and can even lead to increased production of toxic metabolites (Table **1**).

## CONCLUSION

Neurodegenerative diseases, such as AD and PD, present profound challenges to an individual’s quality of life, primarily due to their progressive nature and the current lack of disease-modifying treatments. Despite extensive research efforts, the underlying molecular mechanisms are still not completely understood. However, a multifactorial approach targeting various pathways implicated in neurodegeneration offers promise. There is a growing interest in natural bioactive compounds derived from plants, animals, and fungi, known for their antioxidant and anti-inflammatory properties. In this review, we explored the neuroprotective potential of bioactive compounds extracted from plants possessing sedative and mood-modulating effects in mitigating oxidative stress, inflammation, mitochondrial dysfunction, and aberrant protein folding, which are critical factors involved in the pathogenesis of both AD and PD. This emerging field shows great promise for developing new therapeutic interventions to prevent and/or delay neurodegenerative disorders.

## Figures and Tables

**Table 1:** List of the main bioactive compounds from sedative and mood-modulating plants and their potential beneficial effects.

**Plants**	**Bioactive Compounds**	**Potential Beneficial Effects**	**Results Obtained ** **(Theoretical or ** **Experimental)**	**References**
*Griffonia * *simplicifolia*	Saponins (secondary metabolites) 5-hydroxytryptophan	Antioxidant	Experimental	[67, 68]
*Sambucus * *nigra*	Flavonoids Phenolic Compounds (Hydroxycinnamic acids)	Antioxidant	Experimental	[76]
*Grewia * *tiliaefolia*	Flavonoids (Vitexin)	Antioxidant Anti-aggregating properties of abnormal proteins	Experimental	[78]
*Passiflora * *incarnata*	Flavonoids (Vitexin)	Antioxidant Anti-inflammatory Anti-aggregating properties of abnormal proteins	Experimental	[86, 88, 89]
*Crataegus * *oxyacantha*	Triterpenoid (Oleanolic and Ursolic Acid) Phenolic compounds, Flavonoids	Antioxidant	Theoretical/ Experimental	[95-98]
*Valeriana * *officinalis*	Valerenic Acid, Valerenal and Valepotriates (secondary metabolites) Flavonoids	Antioxidant Anti-inflammatory Anti-aggregating properties of abnormal proteins Restoration of Mitochondrial Function	Experimental	[105-107]
*Rhodiola * *rosea*	Glycoside of Tyrosol (Rhodioloside) Phenolic Compounds (Rosavins) Phenolic Compounds (P-tyrosol)	Antioxidant Anti-inflammatory	Experimental/ Theoretical	[108-110]
*Humulus * *lupulus*	Prenylated flavonoid (Xanthohumol; 8-Prenylnaringenin) Phenolic Compounds (Flavan-3-ol Glycosides) Polyphenols (Procyanidins)	Antioxidant Anti-inflammatory Anti-aggregating properties of abnormal proteins	Theoretical/ Experimental	[114, 115]
*Chamomilla matricaria*	Sesquiterpene alcohol (α-Bisabolol) Sequiterpene (Camazulene) Sequiterpene oxide (Bisabolol Oxide A) Hydroxycinnamic acid (Chlorogenic Acid) Flavonoids (Apigenin-7-glucoside, Rutin, cynaroside, Luteolin, apigenin and derivatives of Apigenin-7-glucoside)	Antioxidant Anti-inflammatory	Experimental	[120, 121]
*Melissa * *officinalis*	Polyphenolic Compound Flavonoids	Antioxidant Anti-inflammatory	Experimental	[126-128]
*Curcuma * *longa*	Polyphenolic Compound (Curcumin)	Antioxidant Anti-inflammatory Anti-aggregating properties of abnormal proteins Restoration of Mitochondrial Function	Theoretical/ Experimental	[135-137]

## LIST OF ABBREVIATIONS

AD: Alzheimer’s Disease
PD: Parkinson’s Disease
ROS: Reactive Oxygen Species
RNS: Reactive Nitrogen Species
CNS: Central Nervous System
GPX: Glutathione Peroxidase
SOD: Superoxide Dismutase
GSH: Glutathione Reductase
MAO: Monoamine Oxidase
AChE: Acetylcholinesterase
BChE: Butyrylcholinesterase
TNF-α: Tumor Necrosis Factor α
iNOS: Induced Nitric Oxide Synthase
LPS: Lipopolysaccharides
